# Severe Mycoplasma pneumoniae Pneumonia During the 2023–2024 European Re-Emergence: Why Severity Does Not Predict Macrolide Resistance

**DOI:** 10.3390/antibiotics15050524

**Published:** 2026-05-21

**Authors:** Enrico Perugini, Ludovica Ferrari, Marco Iannetta, Barbara Bartolini, Valentina Dimartino, Marco Favaro, Carla Fontana, Loredana Sarmati

**Affiliations:** 1 Infectious Diseases Unit, Department of System Medicine, Tor Vergata University, 00133 Rome, Italy; enrico.perugini.1995@gmail.com (E.P.); marco.iannetta@uniroma2.it (M.I.); sarmati@med.uniroma2.it (L.S.); 2 Department of Infectious Diseases, Galliera Hospital, 16128 Genoa, Italy; ludovica.ferrari@galliera.it; 3 National Institute for Infectious Diseases “Lazzaro Spallanzani”—IRCCS, 00149 Rome, Italy; barbara.bartolini@inmi.it (B.B.); carla.fontana@inmi.it (C.F.); 4 Department Experimental Medicine, University of Rome “Tor Vergata”, 00133 Rome, Italy; favaro@uniroma2.it

**Keywords:** *Mycoplasma pneumoniae*, macrolide-resistant mutations, real-time PCR, severe pneumonia, antimicrobial stewardship

## Abstract

Background: Following a significant decline during the 2020–2021 SARS-CoV-2 pandemic, *Mycoplasma pneumoniae* (MP) experienced a resurgence across Europe in 2023–2024. Although macrolide-resistant MP has increased globally, severe disease can occur even in the absence of resistance, which highlights the importance of rapid molecular characterization for clinical purposes. In this context, clinical severity is often improperly used as a surrogate marker of macrolide resistance, potentially driving unnecessary antibiotic escalation. Methods: We report a severe MP pneumonia occurring during the 2023–2024 resurgence and evaluate macrolide resistance through a rapid two-step workflow (Real Time-PCR screening for A2063G/A2064G followed by confirmatory 23S rRNA sequencing), to assess whether severity predicts resistance and to support antibiotic stewardship. Results: The patient developed acute hypoxic respiratory failure (PaO_2_ 54.9 mmHg; P/F ratio 110), extensive centrilobular micronodules on chest CT imaging, significant systemic inflammation and elevated liver enzymes. Respiratory support was escalated from a Venturi mask to a high-flow nasal cannula and BiPAP. MP infection was confirmed by multiplex Real Time-PCR (RT-PCR) and supported by positive IgM/IgG serology. RT-PCR targeting A2063G/A2064G mutations revealed no resistance-associated variants, and Sanger sequencing of an 807 bp 23S rRNA fragment confirmed a wild-type genotype. Despite severe hypoxemic respiratory failure, no resistance-associated variants were detected, documenting a clear severity–genotype mismatch. Clinical and radiological improvement followed second-line antibiotic therapy. Conclusions: Severe MP pneumonia can occur despite the absence of macrolide resistance. During MP re-emergence, clinical severity should not be used to infer macrolide resistance. Integrating nucleic acid amplification test (NAAT) diagnosis with rapid genotyping/confirmatory 23S rRNA sequencing can prevent misclassification, reduce unwarranted broad-spectrum escalation, and strengthen antimicrobial stewardship decisions.

## 1. Introduction

*Mycoplasma pneumoniae* (MP) is one of the leading causes of community-acquired pneumonia (CAP) in children, adolescents, and young adults worldwide [1,2]. Following a marked decline in pathogen circulation during the COVID-19 pandemic, a significant resurgence of MP has been reported in Europe since mid-2023, affecting otherwise healthy individuals [3,4]. Macrolides remain the recommended first line therapy for MP infections; however, a global increase in macrolide-resistant MP (MRMP) strains over the past two decades has raised concern regarding empirical treatment strategies and therapeutic failure [5,6]. Notably, severe pneumonia has also been described in patients infected with macrolide-susceptible strains, indicating that disease severity is not exclusively driven by antimicrobial resistance, but is strongly influenced by the host immune response [7,8,9]. Excessive activation of macrophages and T lymphocytes, with subsequent release of pro-inflammatory cytokines, may lead to a dysregulated immune response and significant tissue damage [10], even in the presence of antibiotic susceptibility. This immunopathogenic process is associated with peri bronchial and interstitial inflammatory infiltrates and can substantially contribute to clinical deterioration. Against this background, molecular characterization of circulating MP strains is essential to accurately distinguish macrolide-susceptible from macrolide-resistant isolates, supporting targeted therapeutic decisions and improved interpretation of the clinical course. Therefore, severe MP pneumonia in otherwise healthy young adults represents a clinically relevant scenario that challenges the disease severity paradigm centered on resistance. In routine practice, severe presentation is frequently interpreted as indirect evidence of macrolide resistance, prompting early escalation to second-line regimens. However, this heuristic may be misleading during the current European resurgence, with relevant implications for antimicrobial stewardship and for interpreting treatment response. Here, we describe a severe MP pneumonia in a previously healthy young adult and use rapid 23S rRNA genotyping to demonstrate that severity does not necessarily predict macrolide resistance, highlighting a practical diagnostic–therapeutic framework to support stewardship.

## 2. Results

### 2.1. Clinical Data and Case Definition

**Figure 1:**
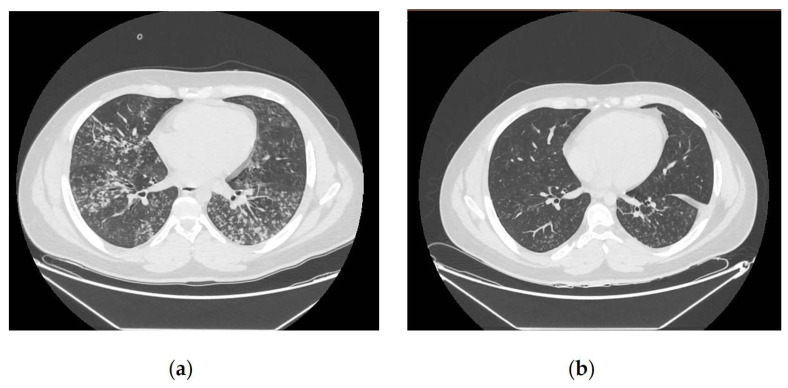
Chest CT findings at admission and follow-up: (**a**) axial CT images obtained on admission show diffuse centrilobular micronodules involving the middle lobe, lingula, and basal segment of the left lower lobe, consistent with atypical pneumonia; (**b**) follow-up CT performed on day 13 demonstrates marked reduction in micronodular opacities and partial re aeration of previously affected regions.

**Figure 2:**
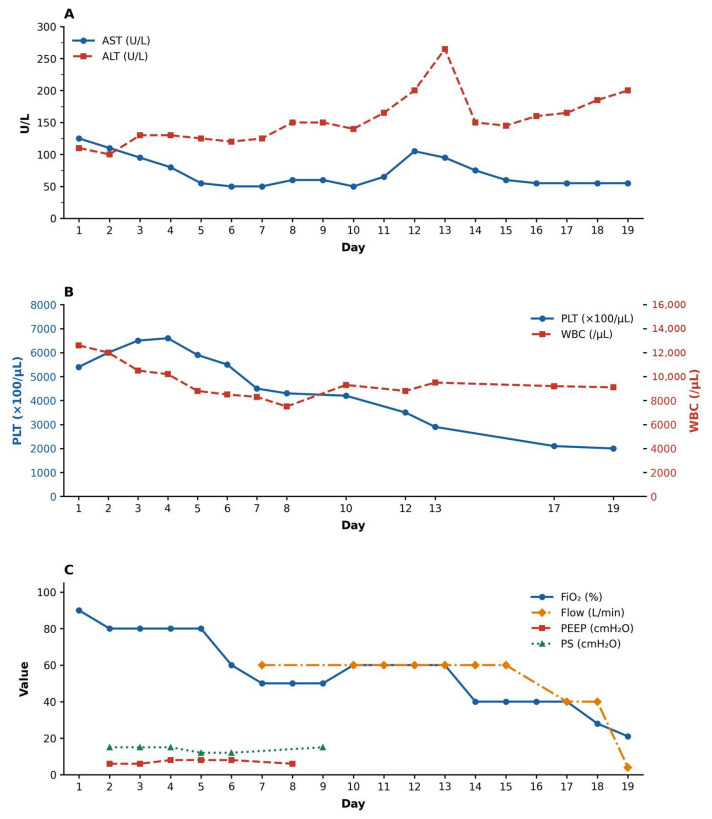
Longitudinal laboratory and respiratory support trends during hospitalization. Time series plots showing: (**A**) evolution of AST and ALT levels; (**B**) platelet count and white blood cell count trajectories; (**C**) respiratory support parameters, including fraction of inspired oxygen (FiO_2_), positive end-expiratory pressure (PEEP), pressure support (PS), and bilevel positive airway pressure (BiPAP). Parameters improve progressively in parallel with clinical recovery.

An 18-year-old male in previously good health presented with cough, fever and dyspnoea. Arterial blood gas analysis on room air revealed severe hypoxaemia (PaO_2_ 54.9 mmHg; P/F ratio 110) and mild respiratory alkalosis (pH 7.52, pCO_2_ 28.4 mmHg). Admission laboratory evaluation revealed leukocytosis (12,600 cells/μL), with a predominance of neutrophils (9880 cells/μL), thrombocytosis (539,000 cells/μL), and markedly elevated C-reactive protein (175 mg/L). There was also an increase in transaminases (AST 121 U/L and ALT 109 U/L). Chest computed, tomography revealed diffuse centrilobular micronodules in the middle lobe, the lingula and the basal segment of the left lower lobe, which are consistent with atypical pneumonia. Respiratory support was rapidly escalated from a Venturi mask to a high-flow nasal cannula, and subsequently to non-invasive ventilation with BiPAP. Based on clinical assessment, corticosteroid therapy with dexamethasone was initiated at 4 mg/day for the first 3 days, followed by 6 mg/day, for a total duration of 2 weeks. Anticoagulation with enoxaparin 4000 IU/day was also started. Given the ongoing resurgence and concerns about MRMP, the initial severity also raised the possibility of macrolide resistance, informing early therapeutic escalation pending genotypic confirmation. Empirical antibiotic therapy consisted of levofloxacin 500 mg every 12 h and doxycycline 100 mg every 12 h, both administered for 2 weeks. During hospitalization, respiratory and laboratory parameters improved progressively. Liver enzymes temporarily increased, peaking around day 12, before returning to normal levels. This pattern is consistent with the reported extrapulmonary manifestations of *Mycoplasma pneumoniae* infection, or a transient drug-related effect. A follow-up chest CT scan performed on day 13 showed a significant reduction in the size and density of centrilobular micronodules, with partial re-aeration of previously affected lung segments (Figure 1). A progressive reduction in oxygen requirements and ventilatory pressures occurred alongside the normalization of inflammatory and hematological markers (Figure 2). The patient was discharged on day 20 after full recovery of respiratory function.

### 2.2. Laboratory Investigations

**Figure 3:**
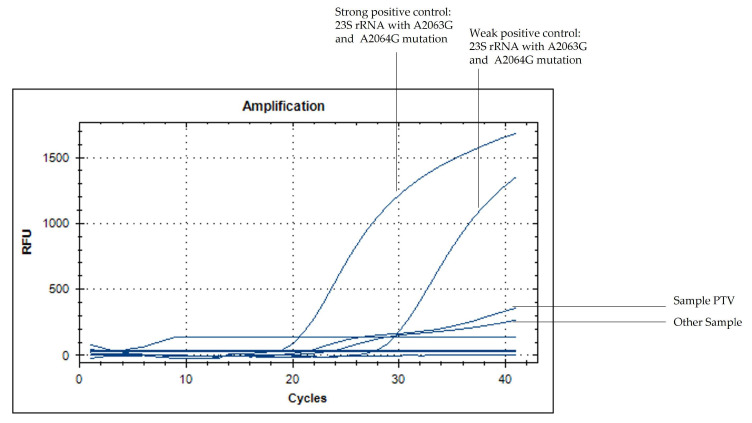
Molecular assessment of macrolide resistance mutations in the 23S rRNA gene. Real time PCR amplification curves targeting A2063G and A2064G mutations show no detectable resistant signal in the patient sample. Sequencing of the 807 bp 23S rRNA fragment confirms a wild-type genotype, ruling out macrolide resistance-associated substitutions.

Multiplex RT-PCR performed on sputum identified *Mycoplasma pneumoniae* DNA, and serology results supported an acute infection (IgM 5.9 UR/mL; IgG 196 UR/mL). Due to global concerns regarding macrolide-resistant MP the empirical antimicrobial therapy was optimized early on to include doxycycline and levofloxacin. Targeted molecular analysis showed no evidence of macrolide resistance; RT-PCR was negative for the A2063G and A2064G mutations (Figure 3), and Sanger sequencing of an 807 bp fragment of the 23S rRNA gene confirmed a wild-type genotype. Overall, molecular testing consistently supported a macrolide-susceptible (wild-type) genotype despite severe disease.

## 3. Discussion

The re-emergence of *Mycoplasma pneumoniae* (MP) in Europe in 2023–2024 is important for understanding severe infections in young, otherwise healthy individuals. This case confirms that severe MP pneumonia is not exclusively associated with macrolide-resistant strains, since the identified isolate carried a fully susceptible 23S rRNA genotype. The discrepancy between clinical severity and macrolide susceptibility indicates a significant role of host-mediated inflammatory mechanisms, which is consistent with previous reports describing cytokine-driven pulmonary injury in MP infections [7,9,11]. A key practical implication is that disease severity alone should not be used as an indicator of resistance. Importantly, this observation does not challenge current recommendations for prompt empirical antibiotic therapy in severe cases. Rather, it aims to support the optimization of these recommendations through early diagnostic clarification, including rapid molecular testing and resistance genotyping.

During periods of heightened alertness for MRMP, this assumption may lead clinicians to prescribe broader regimens more frequently, thereby increasing exposure to fluoroquinolones or tetracyclines, even when macrolide susceptibility is retained. Phenotypic antimicrobial susceptibility testing was not performed because culture-based assays for MP are not routinely available in real-time clinical practice and would not support timely therapeutic decisions.

Rapid genotyping is therefore valuable not only for surveillance, but also for real-time stewardship decisions. The radiological findings observed at presentation, which included confluent centrilobular micronodules involving multiple lobes, together with the rapid improvement seen on follow-up imaging, support the diagnosis of *Mycoplasma pneumoniae* (MP) pneumonia, despite macrolide susceptibility, which is characterized by a marked inflammatory component. The close temporal association between clinical stabilization, reduction in ventilatory support, normalization of laboratory parameters and radiological resolution highlights the dynamic and reversible nature of the inflammatory process (see Figure 1 and Figure 2). Combining confirmatory genotyping, which enabled rapid identification of the cause, and multiplex RT-PCR also allowed an accurate assessment of macrolide susceptibility.

Although global epidemiological data indicates a significant increase in the prevalence of macrolide-resistant *Mycoplasma pneumoniae* (MRMP), with marked heterogeneity across Europe, molecular confirmation of antibiotic susceptibility remains essential to avoid misclassification and the unnecessary escalation of antimicrobial therapy. In this case, escalation to doxycycline and levofloxacin was justifiable while MRMP could not be excluded. However, subsequent confirmation of a wild-type 23S rRNA genotype demonstrated how genotyping can rapidly reorient therapeutic rationale, thus preventing resistance misclassification driven solely by clinical severity. Overall, this case supports the practical diagnostic and therapeutic approach of combining syndromic molecular tests (NAATs) with rapid resistance genotyping or confirmatory sequencing for the current resurgence of MP infection. This indicates that clinical severity and antibiotic resistance do not necessarily coincide, so stewardship decisions should be based not only on clinical impression, but also on the infectious agent’s genotype.

This report is limited by its single case nature, which precludes generalization; however, the detailed clinical, radiological, and molecular characterization strengthens the diagnostic and antimicrobial stewardship message in the context of the current MP resurgence, without questioning the validity of existing therapeutic guidelines, but rather emphasizing the importance of early diagnostic clarification to optimize treatment decisions.

## 4. Materials and Methods

Clinical, laboratory, and radiological data were collected from hospital admission through discharge, including arterial blood gas analyses, complete blood count, inflammatory markers, liver enzymes, and respiratory support parameters. Imaging included chest computed tomography performed on admission and during follow-up. Etiological testing was carried out using a multiplex RT-PCR panel for respiratory pathogens on a sputum sample, followed by serological assays for *Mycoplasma pneumoniae* IgM and IgG. Macrolide-resistance was evaluated using a two-step molecular workflow. First, RT-PCR screening targeted the A2063G and A2064G mutations of the 23S rRNA gene using a commercial assay, with the *Mycoplasma Pneumoniae* and Macrolides-Resistant Strain Nucleic Acid Test Kit (Jiangsu Mole Bioscence Co. Taizhou, Jiangsu Province, China). The analysis was extended with Sanger sequencing for the genomic portion of 23S, potentially containing the mutations known in the literature. The RT-PCR was performed on a GeneAmp PCR System 9700 thermocycler (Applied Biosystems, Foster City, CA, USA) under the following conditions: enzymatic activation at 95 °C for 1 min, followed by 30 denaturation steps at 95 °C for 15 s and annealing/extension at 60 °C for 30 s, with the latter two steps repeated for a total of 35 cycles. The resulting RT-PCR amplicon was 807 bp in length (forward primer 5′-GAACGGCGGCCGTAACTATA-3′; reverse primer 5′-GGCGCTACAACTGGAGCATA-3′). Sequencing was performed on a 3500 Dx Genetic Analyzer (Applied Biosystems, Thermo Fisher Scientific, Waltham, MA, USA). Sequence analysis was carried out using BioEdit software version 7.0.5.3 and revealed no mutations in the region of interest. Molecular assays were performed on available clinical specimens using the appropriate controls in accordance with routine diagnostic practices.

## 5. Conclusions

This case demonstrates that severe, macrolide-susceptible pneumonia associated with the wild-type 23S rRNA genotype can occur in otherwise healthy young adults. During the current resurgence, it is important to note that clinical severity should not be used to infer macrolide resistance. An approach combining syndromic NAAT with rapid resistance genotyping and 23S rRNA sequencing can prevent misclassification based on severity, support rational antibiotic selection, and avoid unnecessary escalation to broad-spectrum antibiotics. However, as this is a single-case report, it is not possible to estimate the proportion of similar cases among hospitalizations related to pneumonia.

## Data Availability

The data presented in this study are available on request from the corresponding author.

## Abbreviations

The following abbreviations are used in this manuscript:

MP	*Mycoplasma pneumoniae*
CAP	Community-acquired pneumonia
MRPM	Macrolide-resistant MP
AST	Aspartate aminotransferase
ALT	Alanine aminotransferase
CT	Computed tomography
WBC	White blood cell
PLT	Platelet
FiO_2_	Fraction of inspired oxygen
PEEP	Positive end expiratory pressure
PS	Pressure support
BiPAP	Bilevel positive airway pressure
